# Intra-subject approach for gait-event prediction by neural network interpretation of EMG signals

**DOI:** 10.1186/s12938-020-00803-1

**Published:** 2020-07-28

**Authors:** Francesco Di Nardo, Christian Morbidoni, Guido Mascia, Federica Verdini, Sandro Fioretti

**Affiliations:** 1 Department of Information Engineering, Università Politecnica delle Marche, via Brecce Bianche, 60131 Ancona, Italy; 2 Laboratory of Bioengineering and Neuromechanics of Movement, University of Rome “Foro Italico”, P.zza Lauro de Bosis 6, 00135 Rome, Italy

**Keywords:** Surface EMG, Machine learning, Neural networks, Gait-phase classification, Ground walking, Intra-subject data

## Abstract

**Background:**

Machine learning models were satisfactorily implemented for estimating gait events from surface electromyographic (sEMG) signals during walking. Most of them are based on inter-subject approaches for data preparation. Aim of the study is to propose an intra-subject approach for binary classifying gait phases and predicting gait events based on neural network interpretation of sEMG signals and to test the hypothesis that the intra-subject approach is able to achieve better performances compared to an inter-subject one. To this aim, sEMG signals were acquired from 10 leg muscles in about 10.000 strides from 23 healthy adults, during ground walking, and a multi-layer perceptron (MLP) architecture was implemented.

**Results:**

Classification/prediction accuracy was tested vs. the ground truth, represented by the foot–floor-contact signal provided by three foot-switches, through samples not used during training phase. Average classification accuracy of 96.1 ± 1.9% and mean absolute value (*MAE*) of 14.4 ± 4.7 ms and 23.7 ± 11.3 ms in predicting heel-strike (HS) and toe-off (TO) timing were provided. Performances of the proposed approach were tested by a direct comparison with performances provided by the inter-subject approach in the same population. Comparison results showed 1.4% improvement of mean classification accuracy and a significant (*p *< 0.05) decrease of *MAE* in predicting HS and TO timing (23% and 33% reduction, respectively).

**Conclusions:**

The study developed an accurate methodology for classification and prediction of gait events, based on neural network interpretation of intra-subject sEMG data, able to outperform more typical inter-subject approaches. The clinically useful contribution consists in predicting gait events from only EMG signals from a single subject, contributing to remove the need of further sensors for the direct measurement of temporal data.

## Background

Gait analysis is acknowledged as the main approach for quantitatively assessing the alteration of motor function in different contexts, such as in basic research and clinics. Technological development is making available smart and wearable sensors (inertial measurement units, IMUs) and robust artificial intelligence methods for handling large amount of data and signals (machine and deep learning). This kind of innovation is starting to allow a reduction of the complexity of experimental protocols for gait analysis and a cheaper, less-invasive, and more comfortable assessment of gait data. This is particularly true for the problem of assessing gait temporal parameters and events, such as stride length and the timing of heel-strike (i.e., the instant when the foot touches the ground) and toe-off (i.e., the instant when the foot-toes clear the ground). In many instances, IMUs showed to be convenient and reliable for an ecological (out of the laboratory) assessment of walking parameters [1]. However, it has been reported that EMG-based approach seems to be preferable over different approaches (including IMU), for specific environments such as control of exoskeleton devices [2], where EMG signals could be used to identify segment motion in advance, thus limiting delays in control action. An example of this is reported by Wentink et al. [3] who showed that the analysis of EMG signals allows assessing gait initiation earlier (63–138 ms) than inertial sensors, in a population of transfemoral amputees. In this and other cases, EMG-based approach seems to be really valuable [4–6]. Moreover, in the analysis of the majority of neuromuscular diseases, such as for example spastic cerebral palsy, the acquisition of sEMG signals is essential. Thus, the possibility of assessing HS and TO timing directly from sEMG signal, without using additional sensors such as foot-switch and IMUs, seems to be very useful in those cases.

Machine learning approaches were also satisfactorily implemented for the estimation of gait events from both kinematic data [2, 7–9] and electromyographic (EMG) signals [9–12] during walking. The success of machine learning approaches has opened a novel perspective for reducing the complexity of experimental set-up. Predicting gait events from only EMG signals could remove the need of further sensors or systems (foot-switch sensors, pressure mats, IMUs, stereo-photogrammetry [1, 13–15]) for the direct measurement of temporal data. This would be particular suitable for specific fields where measuring myoelectric signals is strongly recommended, such as the analysis of neuromuscular diseases or for walking-aid devices [16, 17].

Only few efforts in this direction [11, 12] proved to be able in providing a reliable classification of swing and stance phases and an accurate prediction of heel-strike (HS) and toe-off (TO), with mean errors comparable to those reported for IMU-based studies [1]. Nazmi et al. [11] fed EMG-based features to a single hidden layer neural network, reporting for unseen subjects a mean classification accuracy of 77% and mean absolute error of 35 and 49 ms in assessing HS and TO, respectively. The present group of researchers interpreted EMG signals by means of a multi-layer perceptron classifier with the aim of predicting HS and TO [12]. Outcomes on unseen subjects were promising, showing a mean classification accuracy of 93.4% and a mean accuracy error of 21 ms for HS and 38 ms for TO. These studies are based on the so-called inter-subject approach for data preparation, which consists in training the neural network with EMG data acquired during different strides of a population of homogeneous subjects and then testing the network on a population of brand new subjects [11, 12]. Moreover, to measure classification performances also for learned subjects (i.e., subject included only in training phase), the training phase was further split into two subsets: training set containing the first part of each subject signal and the test set including brand new strides (not used for training) taken from the subjects involved in training. Both studies indicated that classification performances were better for learned subjects than for unseen ones, in terms of accuracy (+ 10% in [11] and + 1.5% in [12]) and standard deviations (SD). Larger variability reported for unseen subjects seems to indicate that walking patterns could be very different from subject to subject, making the classification harder in subjects never seen before.

All these results and considerations raise the issue if a totally intra-subject approach could drive to a better performance in gait-phase classification. With intra-subject approach, we mean training the neural network with EMG data acquired during different strides of a single subject and then testing the network on brand new strides of the same subject. The few and preliminary studies reporting classification results based on the intra-subject approach, including our own, seem to support this hypothesis [10, 18–22]. Meng et al. [10] applied hidden Markov models to EMG signal to identify stance and swing phases, reaching a classification accuracy of 91%. Ziegier et al. [19] reported a best-case accuracy of around 96% in the classification of gait phases by training a support vector classifier with EMG signals. To the same purpose, Joshi et al. [20] used Bayesian Information Criteria along with some standard feature extraction methods and Linear Discriminant Analysis classification algorithm. Best individual accuracies were around 94%. Population involved in these three preliminary studies included two subjects at most. Also our preliminary reports showed encouraging results, characterized by mean intra-subject classification accuracy of 95.2 ± 1.6% [18]. None of the above-mentioned studies [10, 18–20] attempted to assess gait events.

Thus, the purpose of the present study is (1) to propose an intra-subject approach for binary classification and gait-event prediction; (2) to test the hypothesis that an intra-subject approach is able to achieve better performances in gait phase classification compared to an inter-subject one and to extend this hypothesis to gait-event prediction. To this aim, an intra-subject approach was implemented for neural network interpretation of 10 surface electromyographic (sEMG) signals collected in 23 subjects. To test our hypothesis, a direct comparison was performed with classification and prediction performances provided by the inter-subject approach in the same population.

## Results

**Fig. 1:**
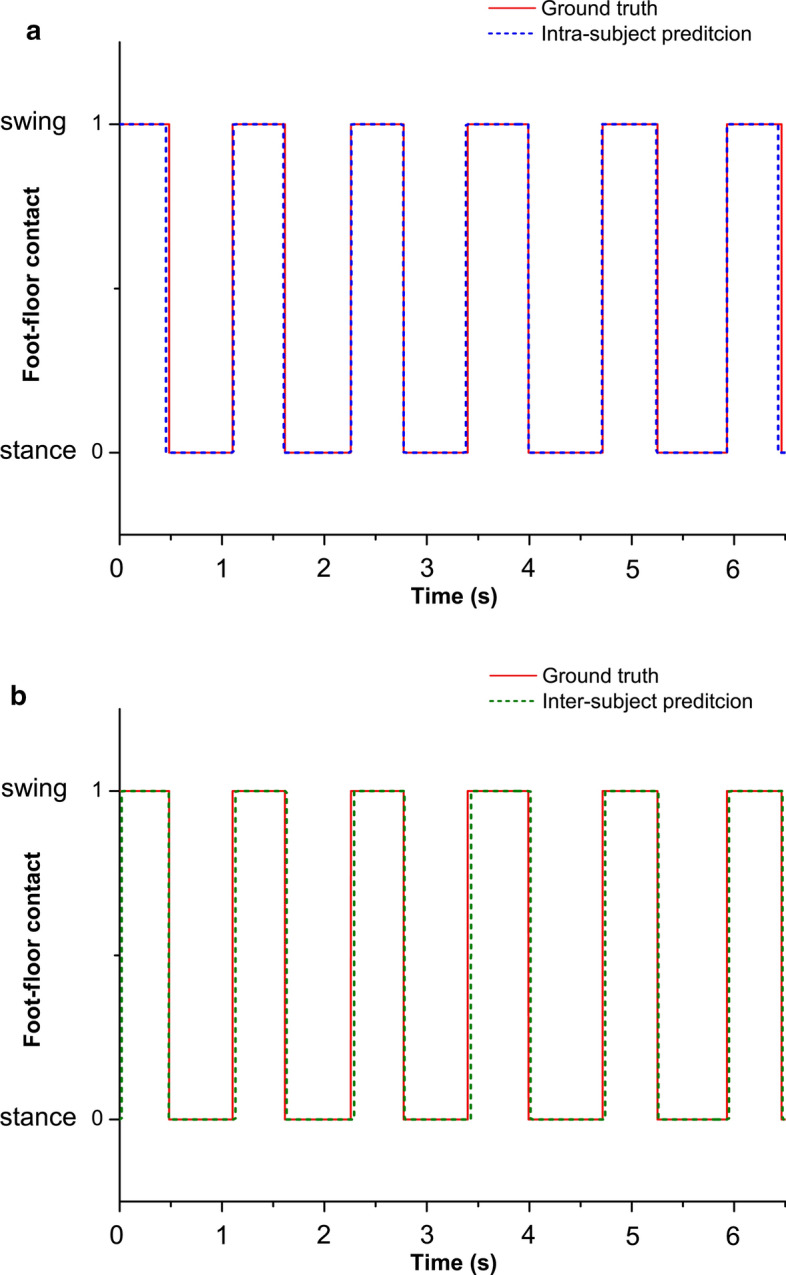
Example of predictions of foot–floor-contact signal in the same six strides of a representative subject (subject 8), provided by intra (**a**) and inter (**b**) subject approaches

The inter-subject approach was considered as the benchmarking approach, since it was validated in [12]. In the present study, it was applied to 10 sEMG signals (five per leg) acquired from 23 adults. The inter-subject approach implemented here in unseen subjects improves the performances reported in [12] both in terms of classification accuracy, 94.7 ± 2.3% vs. 93.4 ± 2.3%, and prediction error, *HS*-*MAE*: 18.7 ± 7.8 ms vs. 21.6 ± 7.0 ms; *TO*-*MAE*: 35.1 ± 20.0 ms vs. 38.1 ± 15.2 ms. Moreover, accuracy in each single subject does not fall below 90%, except for a single subject (subject 4, 88.9%). Best-case accuracy is 98.0% (subjects 18 and 23). Mean inter and intra-subject accuracies of gait-phase classification were compared in every subject in Table 1. Mean values over population (23 subjects) are shown in the last row of the table. The direct comparison between mean values showed an improvement of 1.4 points of the classification accuracy provided by the intra-subject approach (96.1 ± 1.9%), compared with the inter-subject approach (94.7 ± 2.3%). Besides gait-phase classification, the present study also provides a prediction of foot–floor-contact signal and a consequent assessment of HS and TO events. An example of predictions of foot–floor-contact signal provided by both inter and intra-subject approaches is reported in Fig. 1. Detailed comparison of the performance achieved with inter and intra-subject approaches is reported in Tables 2 and 3 for HS and TO prediction, respectively. A significant mean reduction of mean absolute error (*MAE*) was detected in the prediction of HS provided by the intra-subject approach, compared with the inter-subject approach (18.7 ± 7.8 ms vs. 14.4 ± 4.7 ms; 23% reduction, *p* < 0.05). In the same way, a significant mean reduction of *MAE* was detected in the prediction of TO provided by the intra-subject approach, compared with the inter-subject approach (35.1 ± 20.9 ms vs. 23.7 ± 11.3 ms; 33% reduction, *p* < 0.05). No further significant differences were detected between groups.

**Table 1:** Stance vs. swing classification accuracy provided by inter and intra-subject approach

Subject	Classification accuracy (%)			
	Inter-subject		Intra-subject	
	Train	Test	Train	Test
1	96.9	97.7	97.5	97.5
2	96.0	94.4	97.9	96.8
3	95.6	94.8	96.5	95.7
4	96.9	88.9	95.1	94.3
5	97.2	93.9	97.8	97.8
6	96.8	94.9	97.4	97.0
7	96.8	95.5	98.1	97.6
8	97.0	94.7	97.9	97.3
9	96.8	94.0	97.1	95.9
10	96.1	92.7	94.8	97.0
11	96.8	92.2	93.0	93.1
12	97.3	92.6	97.5	96.4
13	97.1	94.3	96.2	96.9
14	96.8	94.7	96.5	96.6
15	96.7	94.9	97.2	97.0
16	96.9	96.5	97.9	97.5
17	97.2	93.8	98.2	98.1
18	96.7	98.0	97.5	97.5
19	96.7	97.8	97.9	96.8
20	96.7	97.8	96.5	95.7
21	97.4	91.3	97.1	90.3
22	97.9	94.3	97.3	91.9
23	97.1	98.0	90.8	95.2
Mean ± SD	96.8 ± 0.5	94.7 ± 2.3	96.7 ± 1.8	96.1 ± 1.9

**Table 2:** *MAE* (mean absolute error), precision, recall, and F1-score provided by inter and intra-subject approach for Heel-Strike (HS) prediction

HS	MAE (ms)		Precision		Recall		F1-score	
Subject	Inter	Intra	Inter	Intra	Inter	Intra	Inter	Intra
1	14.7	11.5	1.00	1.00	1.00	1.00	1.00	1.00
2	29.7	17.3	0.99	1.00	0.99	0.99	0.99	1.00
3	19.6	17.4	0.97	1.00	0.99	1.00	0.98	1.00
4	12.8	10.3	1.00	1.00	1.00	0.99	1.00	1.00
5	26.4	8.5	1.00	1.00	1.00	0.99	1.00	0.99
6	11.5	7.4	1.00	1.00	1.00	0.99	1.00	0.99
7	19.0	16.6	1.00	1.00	1.00	0.99	1.00	1.00
8	25.6	16.2	0.99	1.00	1.00	0.99	1.00	1.00
9	19.9	15.5	1.00	1.00	1.00	1.00	1.00	1.00
10	27.5	16.1	0.99	1.00	0.99	0.99	0.99	0.99
11	9.3	9.2	1.00	1.00	0.98	0.98	0.99	0.99
12	8.0	6.8	1.00	1.00	1.00	0.99	1.00	0.99
13	22.0	16.3	1.00	1.00	0.98	0.99	0.99	0.99
14	20.3	15.0	1.00	1.00	1.00	1.00	1.00	1.00
15	19.3	13.1	1.00	1.00	1.00	1.00	1.00	1.00
16	15.3	10.6	1.00	1.00	1.00	1.00	1.00	1.00
17	36.5	9.2	1.00	1.00	1.00	1.00	1.00	1.00
18	10.8	11.5	1.00	1.00	1.00	1.00	1.00	1.00
19	10.8	17.3	1.00	1.00	1.00	0.99	1.00	1.00
20	11.7	17.4	1.00	1.00	1.00	1.00	1.00	1.00
21	21.3	23.1	0.98	0.95	0.97	0.96	0.97	0.96
22	29.3	20.2	1.00	0.99	1.00	0.96	1.00	0.98
23	9.9	24.0	1.00	0.99	1.00	0.99	1.00	0.99
Mean	18.7*	14.4	0.997	0.996	0.996	0.991	0.996	0.993
SD	7.8	4.7	0.008	0.010	0.009	0.011	0.008	0.010

* *p* < 0.05 between inter and intra-subject approach

**Table 3:** *MAE* (mean absolute error), precision, recall, and F1-score provided by inter and intra-subject approach for Toe-Off (TO) prediction

TO	MAE (ms)		Precision		Recall		F1-score	
Subject	Inter	Intra	Inter	Intra	Inter	Intra	Inter	Intra
1	12.3	14.1	1.00	0.99	1.00	1.00	1.00	1.00
2	25.8	21.8	0.97	1.00	0.98	0.98	0.97	0.99
3	27.6	27.6	0.98	0.98	1.00	0.98	0.99	0.98
4	104.2	49.7	1.00	1.00	0.99	0.98	0.99	0.99
5	36.0	16.4	1.00	1.00	1.00	0.99	1.00	0.99
6	44.7	25.3	1.00	1.00	1.00	0.98	1.00	0.99
7	37.6	13.3	1.00	1.00	1.00	0.98	1.00	0.99
8	27.9	12.0	1.00	1.00	1.00	0.99	1.00	0.99
9	45.1	28.4	1.00	1.00	1.00	0.99	1.00	0.99
10	53.4	13.3	1.00	1.00	1.00	0.98	1.00	0.99
11	55.6	52.9	0.94	0.96	0.93	0.93	0.93	0.95
12	65.8	29.6	1.00	0.99	1.00	0.98	1.00	0.98
13	24.1	15.5	0.98	1.00	0.96	1.00	0.97	1.00
14	29.4	19.6	1.00	1.00	1.00	1.00	1.00	1.00
15	40.3	21.1	1.00	1.00	1.00	1.00	1.00	1.00
16	22.7	17.2	1.00	1.00	1.00	1.00	1.00	1.00
17	28.9	11.9	1.00	1.00	1.00	1.00	1.00	1.00
18	12.4	14.1	1.00	0.99	1.00	1.00	1.00	1.00
19	15.7	21.8	1.00	1.00	1.00	0.98	1.00	0.99
20	15.9	27.6	1.00	0.98	1.00	0.98	1.00	0.98
21	38.1	39.7	0.97	0.92	0.96	0.92	0.96	0.92
22	32.6	30.0	0.98	0.99	0.98	0.96	0.98	0.98
23	12.1	23.0	1.00	0.99	1.00	0.98	1.00	0.98
Mean	35.1*	23.7	0.991	0.990	0.990	0.980	0.991	0.985
SD	20.9	11.3	0.008	0.018	0.019	0.020	0.017	0.018

* *p* < 0.05 between inter and intra-subject approach

## Discussion

The present study was designed to test the hypothesis that an intra-subject approach is able to achieve better performances in terms of stance vs. swing classification and HS and TO timing assessment, compared to an inter-subject one. A multi-layer perceptron (MLP) architecture with three hidden layers composed of 512, 256 and 128 neurons, respectively, and a one-dimensional output was implemented to this aim. The intra-subject approach is articulated as follows: training the neural network with sEMG data measured during around 450 strides of one single subject and then testing the network on around 50 brand new strides of the same subject. The procedure was performed ten times, each time using a different slot as test set (tenfold cross-validation). Results were reported as average value over the tenfold. The present approach was able to provide a very precise classification of gait phases (Table 1, column “intra”), represented by mean (± SD) accuracy over 23 subjects of 96.1 ± 1.9% and supported by the fact that accuracy in each single subject does not fall below 90.3% (subject 21). Best-case accuracy is 98.1% (subject 17). Compared with literature [11, 12, 18–20], accuracy outcomes reported here are very encouraging.

The accurate classification ability and the efficient post-processing of model output guaranteed mean prediction, recall, and F1-score values in gait-event assessment very close to 1. Moreover, an average *MAE* over population of 14.4 ± 4.7 ms (Table 2) and 23.7 ± 11.3 ms (Table 3) was detected in the prediction of HS and TO, respectively. To appreciate the quality of these predictions, it is helpful highlighting that associated *MAE*s correspond approximately to 1% and 2% of a gait cycle duration, respectively. Since large variability of the signal to predict (foot–floor contact) is expected to affect the performance of a classifier, an added value is that present results have been achieved in condition of high variability of foot-switch signals. In fact, the eight-shaped path followed by subjects during the experimental procedure is acknowledged to introduce further gait variability, due to curves, reversing, deceleration, and acceleration, with respect to straight or treadmill walking. To the best of our knowledge, only two studies on this issue are available and neither reported such a small prediction error [11, 12]. Moreover, these outcomes are at least comparable with those reported by analogous IMU studies. Very meaningful from this point of view is the study where 17 IMU-based approaches were tested on 35 healthy subjects [1], showing a media time error ranging from 60 to 65 ms and from −25 to 6 ms for HS and TO prediction, respectively, and a 25th–75th percentile error ranging from 40 to 111 ms and from 68 to 120 ms for HS and TO prediction, respectively. A very recent study remarks the same perspective [23], reporting a 17.83-ms HS-MAE and a 26.96-ms TO-MAE during indoor walking. Thus, we may argue that in specific environments, EMG-based approach may be very useful and as much accurate as other techniques.

These promising performances are supposed to be likely due to the peculiarities of the experimental protocol which provides a great amount of data per subject: numerous strides (about 500) and ten different sEMG signals (five muscles per leg). The choice of the model was made based on the analysis we carried out in [12, 18]. The first study [12] compared the classification accuracy on inter-subject data of five different MLP models of increasing complexity in terms of classification. The second study [18] compared the classification accuracy on intra-subject data of the first three MLP models introduced in [12]. In both studies, the neural network with three hidden layers composed of 512, 256 and 128 units was identified as the model achieving the best accuracy on unlearned subjects. That is the model used in the present study. Trying to reduce the complexity of the neural network, an MLP model with a single hidden layer was tested on the intra-subject data. The number of nodes was set at a value (128) lower than the number of inputs (200). Results show that the single-layer model achieves a mean classification accuracy on test set of 95.7 ± 1.6% vs. 96.1 ± 1.9% reported by the chosen model, confirming that also in the present population the 3-hidden-layer model allows obtaining (slightly) better performances.

**Fig. 2:**
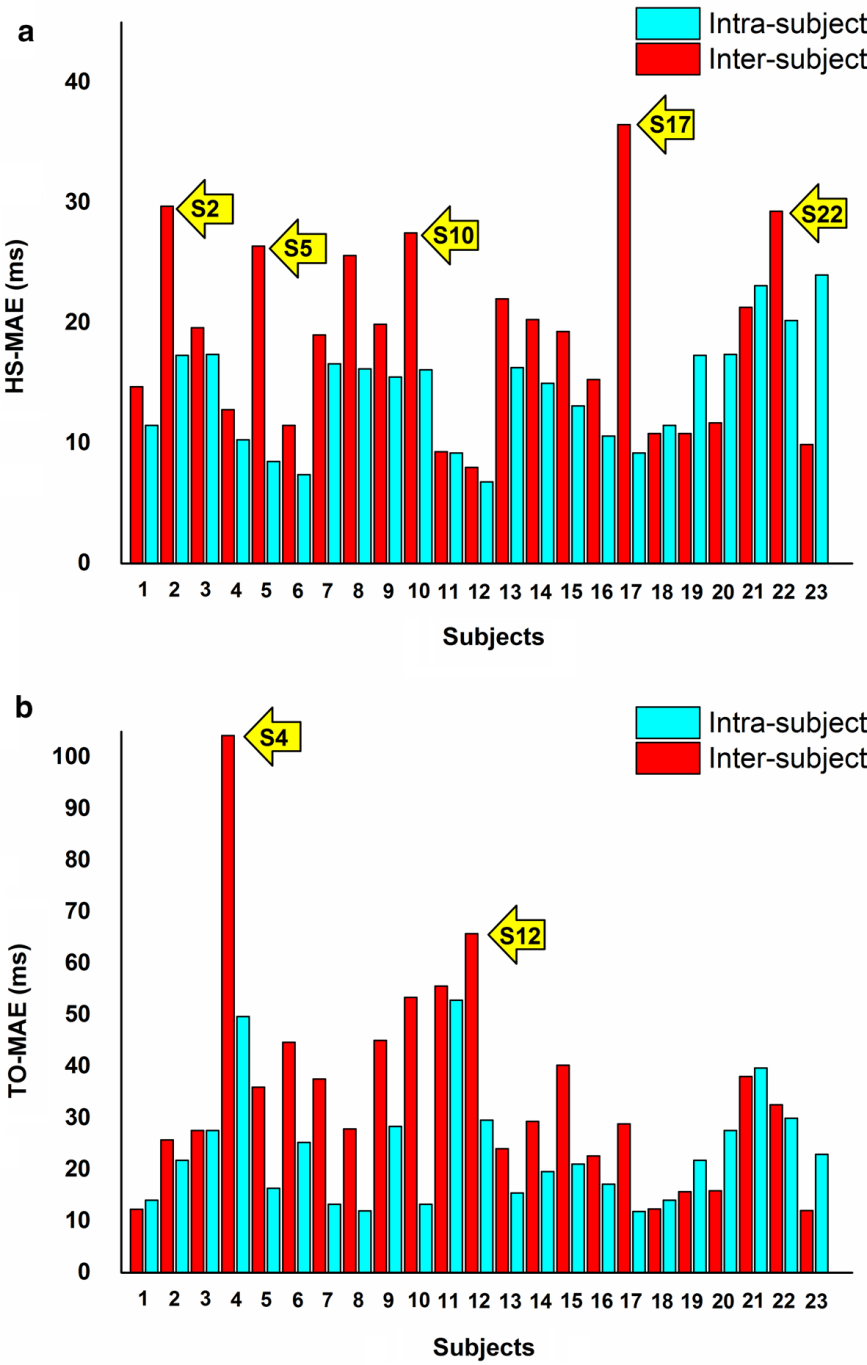
Prediction of HS-MAE (**a**) and TO-MAE (**b**) in each single subject provided by inter-subject (red bars) and intra-subject approaches (cyan bars). Yellow arrows indicate inter-subject predictions which diverge more evidently from the mean values

The robustness of MLP models for gait-phase classification has been previously validated for the inter-subject approach [12]. Thus, the present intra-subject approach was tested by a direct comparison with the performances achieved by the inter-subject one with the same model in the same population. It is worth noticing that, even if in the present study the neural network is the same used in [12], results in both intra-subject and inter-subject are completely new because (1) the training phase was performed including ten sEMG signals (i.e., 5 muscles per leg) vs. the eight sEMG signal used in [12], where RFs were not included; (2) to our knowledge this is the first attempt to provide HS and TO estimation by means of an intra-subject approach. Moreover, the inter-subject approach implemented in the present study improves already the performances reported in [12]. Comparison between intra-subject and the inter-subject approaches (Tables 2 and 3) highlights two main points: the intra-subject approach allows to achieve (1) an improvement of 1.4% of mean classification accuracy (96.1% vs. 94.7%); and (2) a significant (*p* < 0.05) decrease of mean *MAE* in the prediction of both HS (23% reduction) and TO (33% reduction) timing. Despite this, on average also the inter-subject approach appears to work adequately, especially for HS. However, mean value over population often disguises what really happen in a single specific subject. The reason why a different way was tried, introducing the intra-subject approach, is that sometimes HS and, above all, TO predictions in a single subject are not so accurate with the inter-subject approach. To stress this fact, Fig. 2 has been introduced, comparing the prediction in each single subject provided by the two approaches (inter-subject in red and intra-subject in cyan). Yellow arrows in Fig. 2 indicate inter-subject predictions which diverge more evidently from the mean values (18.7 ms for HS and 35.1 for TO). Some of these outliers present very bad prediction (S17 for HS and S4 for TO above all). This problem does not occur for intra-subject predictions, which spread out around the mean value showing a small dispersion of MAE values. This consideration becomes clearer if the single case is discussed. As clearly highlighted in Fig. 2, subject 4 (S4 yellow arrow in panel B) shows very bad inter-subject TO prediction, where an MAE of 104.2 ms is detected. From the clinical point of view such an error is not acceptable because 104.2 ms is about 10% of gait cycle. Thus, we can say that intra-subject approach fails in the prediction of TO in subject 4. Also for intra-subject approach, TO in subject 4 is one of the hardest to predict, but the MAE remains < 50 ms. In this case, there is 54.5 ms difference between the two approaches (nearly 50% reduction of absolute error). The intra-subject approach has been introduced just to fix situation like this. Similar condition has been detected also in subject 12 (S12 yellow arrow in panel B, TO-MAE = 65.8 ms for inter-subject approach and TO-MAE = 29.6 ms for intra-subject approach, difference = 36.2 ms, 45% reduction of absolute error). Inter-subject HS prediction reports the same problem. Subject 17 (S17 yellow arrow in panel A) shows the worst HS prediction (HS-MAE = 36.5 ms) for inter-subject approach. Intra-subject approach reduces MAE to 9.2 ms (difference = 27.3 ms, 75% reduction of absolute error). The same goes for S2, S5, S10, and S22. In all these cases (at least 7 out of 23), the inter-subject approach seems not to work adequately and the contribution of the intra-subject approach appears to be very valuable.

Cited reports [11, 12] identified more difficulties in detecting toe-offs rather than heel-strikes. Accelerometer-based studies provided the same indication [21]. The inter-subject approach computed in the present study confirms this trend, showing *MAE* value in TO prediction up to 104 ms. The proposed intra-subject approach also shows better performance in detecting heel-strikes rather than toe-offs, but with the advantage of limiting the worst-case *MAE* (subject 11) below 53 ms. Ultimately, all these outcomes work together to argue that intra-subject approach outperforms the inter-subject one for this specific task. Proving this is the actual added value of the present study.

In Tables 2 and 3, a time tolerance T of 600 ms is employed to predict HS and TO (as discussed in “Gait-event identification” section). To provide an indirect comparison with previous studies where different approaches based on kinematic data were presented, an additional evaluation is provided. Following the evaluation approach adopted in [22] for IMU-based prediction, the time tolerance T was set to 50 ms (100 samples), which corresponds to the typical error reported in literature [24]. Average results over the 23 subjects are reported in Table 4, for both HS and TO predictions. As expected, reducing T to 50 ms implies a worsening of precision and recall, but concomitantly it improves *MAE* values, for both HS and TO estimation. However, such results confirm that performances provided by the intra-subject approach are sensibly better than those achieved with the inter-subject approach, both in F1-score and in *MAE*. Furthermore, the percentage of HS detected within 50 ms is still relatively large (95% and 93% for intra-subject and inter-subject approaches, respectively), with an average *MAE* of 13.2 ms (inter-subject) and 10.9 ms (intra-subject). TO predictions are, in general, less accurate. However, when using the intra-subject approach, around 90% are detected within 50 ms with an average *MAE* of 14.6 ms. These results are comparable with those reported for IMU studies [22]. In the present paper, signal windows of 10 ms are considered to feed the neural network in both intra- and inter-subject approaches, according to [12]. Further investigation of parameter tuning (involving signal segmentation and windowing) could be an interesting future direction.

**Table 4:** MAE (mean absolute error), precision, recall, and F1-score provided by inter and intra-subject approach using a time tolerance *T* = 50 ms

Subject	MAE (ms)		Precision		Recall		F1-score	
	Inter	Intra	Inter	Intra	Inter	Intra	Inter	Intra
HS mean	13.2	10.9	0.93	0.95	0.92	0.95	0.93	0.95
SD	5.6	2.5	0.06	0.04	0.06	0.04	0.06	0.04
TO mean	21.8	14.6	0.79	0.89	0.78	0.88	0.78	0.89
SD	8.2	4.0	0.20	0.10	0.20	0.11	0.20	0.10

As introduced above, the clinically useful purpose of the study is the attempt at reducing the complexity of the experimental gait analysis set-up, predicting the foot–floor-contact data from neural network interpretation of EMG signal in a single specific subject. The fewer sensors are used, the easier is to safeguard patient comfort. This is true for both inter-subject and intra-subject approaches. To run the inter-subject approach, a large dataset of EMG and foot–floor-contact signals from many different subjects is needed to train the neural network [12]. When such a dataset is available, classification of a new subject does not require further training (and the use of further sensors, such as IMUs or foot-switch sensors) and the inter-subject approach is preferable. However, this is true for those populations where large amounts of data are available. On the contrary, recruiting an adequate number of subjects to build up the dataset for specific condition, pathology, or dysfunction could be a challenging task. This is particularly true for those disorders which are uncommon or rare. The intra-subject approach has been introduced also to provide an alternative way to face this experimental issue. In this approach, only data from the single subject under examination are necessary. It is true that the neural network should be trained with EMG and basographic data for each new subject, but only once. For all the successive tests, no further training is required and further sensors are not necessary. It is acknowledged that for monitoring their rehabilitation process, different kind of patients (neurological disorders, injuries, aging) should undergo periodical tests. The intra-subject approach appears to be very suitable for these cases, since after a first session where EMG and basographic data have to be acquired and the model has to be trained, all the following tests do not need neither the basographic signal acquisition nor the training of the model, but only the acquisition of EMG signals of the single patient under examination. Moreover, the higher precision in gait-event prediction provided by the intra-subject approach allows to correctly detect the small improvements of the patient performances in temporal parameters during rehabilitation and to handle data where duration of gait phases could be strongly altered (aging, Parkinson’s disease) [25, 26]. In these conditions, the intra-subject approach is probably preferable. In conclusion, intra-subject approach has its pros and cons, but its real added value is that it works just when the inter-subject approach seems to fail, because of bad individual performances or the lack of a large dataset of EMG and foot–floor-contact signals to train the neural network.

The present intra-subject approach could also play a relevant role in the so-called Statistical Gait Analysis (SGA). SGA is a recently developed technique, based on the cycle-dependency of muscular activation, which has already provided promising results published in many studies of the present [13, 27] and other groups of researchers [28, 29]. SGA allows to characterize human walking by means of average sEMG features and spatial–temporal parameters extracted from hundreds of consecutive strides per trial that are recorded and classified according to the sequence of foot–floor contact. Intra-subject approach could contribute in reducing the number of instrumentation worn by the subject and necessary for SGA, removing the need of foot-switches. Further fields of application could be exoskeletons [30], smart prostheses, [31] and functional electronic simulation [32].

From the methodological point of view, future developments of the present study could focus on testing the classification/prediction performance provided by a mixed approach. It could consist in testing the neural network in brand new strides of a subject, after training the neural network with EMG data measured in a population of homogeneous subjects (i.e., with similar characteristics, such as age, weight, and height) which includes also the subject under investigation. For example, the neural network could be trained with the 100% of the signals acquired in 22 subjects and the 90% of the signal acquired in a 23rd subject. Then, the neural network could be tested in the remaining 10% of the signal acquired in the 23rd subject.

For clinical and research purposes, stance phase is typically split in three main sub-phases: heel-strike, flat foot contact, and push off. A further advancement could involve the attempt of classifying stance sub-phases and predicting transition timing between them. Moreover, present results are achieved from data acquired during walking at self-selected speed. The potential effect of gait speed could be also considered, as reported in [22]. This would also be an interesting future direction for the present work, as EMG envelopes show adaptations to different gait speeds.

## Conclusions

The present study proposes an accurate methodology for classifying stance vs. swing and predicting the timing of gait events, based on neural network interpretation of intra-subject sEMG data during walking. The clinically useful contribution of the study consists in predicting gait events from only EMG signals, contributing to remove the need of further sensors for the direct measurement of temporal data. A direct comparison in the same population showed that results of the present approach outperform the outcomes provided by the same network with an inter-subject approach. However, the choice of the suitable approach should be driven not only by network performance but also (mainly) by patient comfort and clinical needs.

## Methodology

### Subjects

The dataset included signals recorded from 23 healthy adults (12 females and 11 males). Mean (± SD) characteristics were age = 23.8 ± 1.9 years; height = 173 ± 10 cm; mass = 63.3 ± 12.4 kg. None of the subjects presented any pathological condition or had undergone orthopedic surgery that might have affected lower limb mechanics. Therefore, subjects with joint pain, neurological pathologies, orthopedic surgery, and abnormal gait were not recruited. Overweight and obese subjects (body mass index > 25) were not recruited because obesity is acknowledged to affect muscle function during walking [33].

### Signal acquisition

**Fig. 3:**
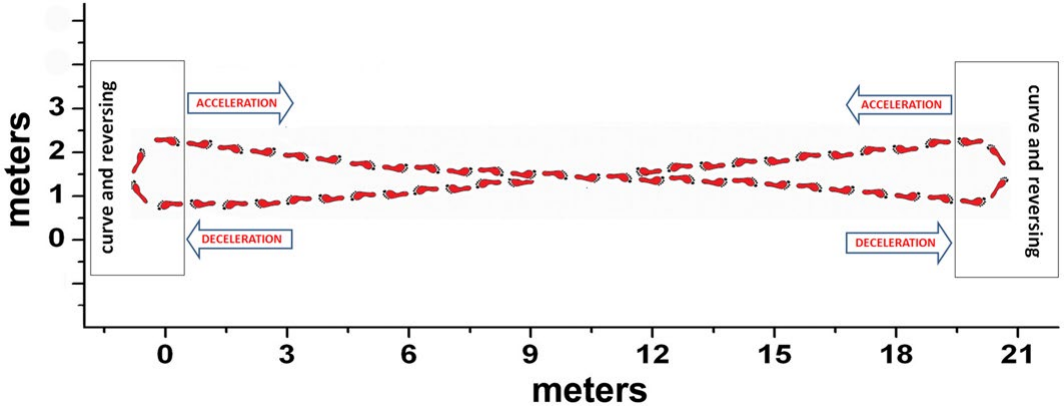
Eight-shaped trajectory followed by each subject during walking

**Fig. 4:**
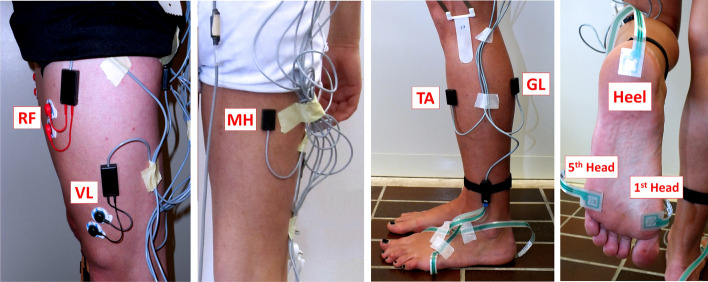
Experimental set-up. sEMG probes are applied over rectus femoris (RF), vastus lateralis (VL), medial hamstrings (MH) tibialis anterior (TA), and gastrocnemius lateralis (GL). Foot-switches are attached under the heel, the first (1st Head) and the fifth (5th Head) metatarsal heads

The multichannel recording system Step32 (Medical Technology, Italy, Version PCI-32 ch2.0.1. DV; resolution: 12 bit; sampling rate: 2 kHz) was employed for the acquisition of all signals. The use of a single system guaranteed the synchronization of all signals. Participants have been instrumented with five sEMG probes and three foot-switches for each leg. Then, they walked with bare feet at self-selected pace (i.e., their own usual, comfortable pace) following an eight-shaped path (Fig. 3), which includes natural deceleration, reversing, curve and acceleration, in the Motion Analysis Lab (Università Politecnica delle Marche, Ancona, Italy), for around 5 min. sEMG signals were registered by means of three single-differential probes with fixed geometry (fixed inter-electrode distance of 8 mm) placed over tibialis anterior (TA), gastrocnemius lateralis (GL), and medial hamstrings (MH) and two single-differential probes with variable geometry (variable inter-electrode distance, starting from a minimum of 12 mm) placed over vastus lateralis (VL) and rectus femoris (RF). Electrode location and orientation were performed under the supervision of a skilled licensed physical therapist, following the SENIAM recommendations [34]. Foot-switches were attached under the heel, the first and the fifth metatarsal heads of each foot for measuring foot–floor-contact signal [27]. Experimental set-up is depicted in Fig. 4. Further details about signal acquisition procedure can be found in [13].

### Signal pre-processing

**Fig. 5:**
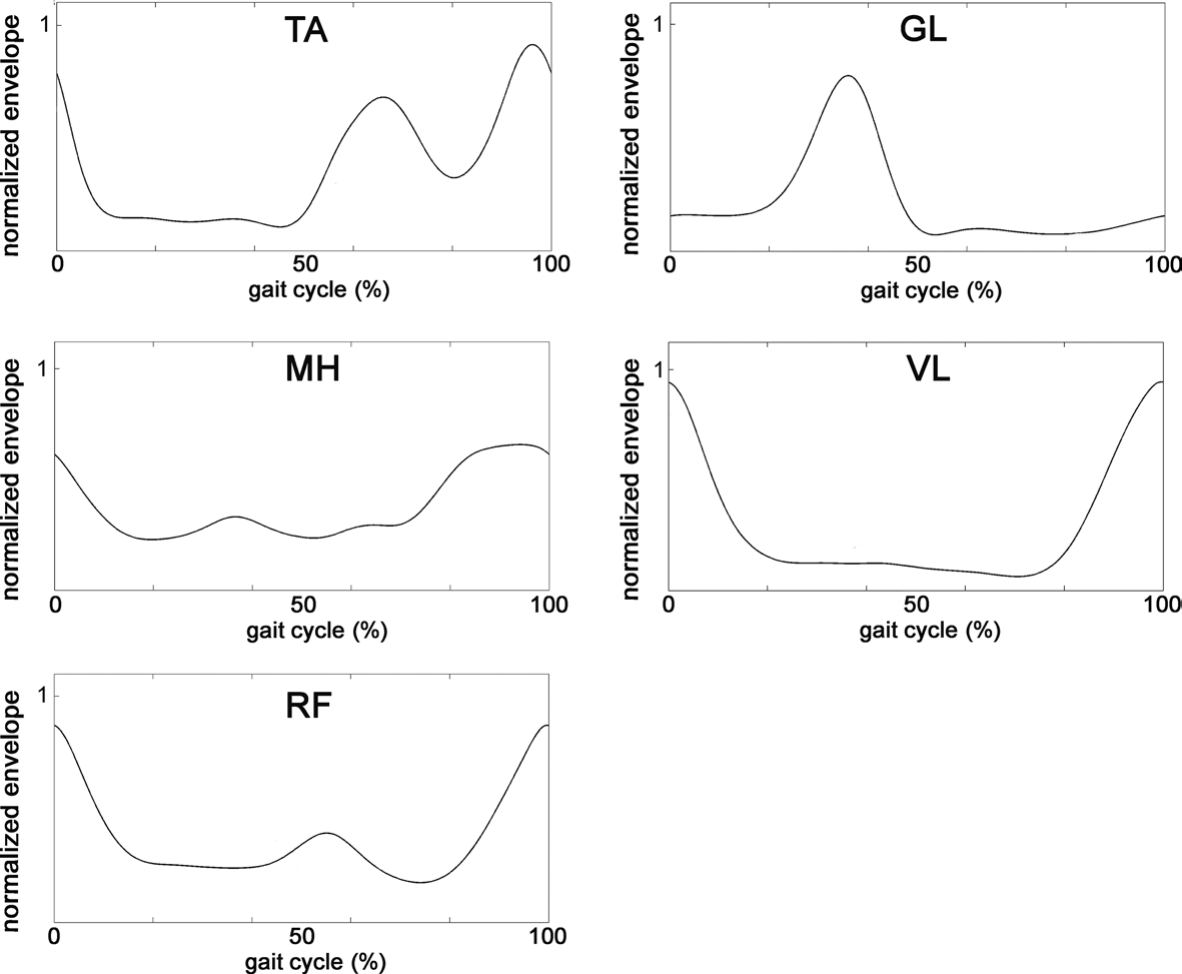
The envelope in a representative stride resulting from the pre-processing of the raw sEMG signals recorded from the five muscles of the right leg

sEMG signals were amplified, high-pass filtered (linear-phase FIR filter, cut-off frequency: 20 Hz) and low-pass filtered (linear-phase FIR filter, cut-off frequency: 450 Hz) for removing motion artifact and high-frequency noise, respectively. Then, the envelope of signal was extracted after a full-wave rectification, applying second-order Butterworth low-pass filter following the classic indication provided by acknowledged studies by Winter et al. [35, 36] and Hermens et al. [34]. Winter proposed a cut-off frequency of 3 Hz [35, 36], while Hermens suggested a cut-off frequency of 10 Hz [34]. In the present paper, a cut-off frequency of 5 Hz was adopted, as a good compromise between the two approaches. Zero-phase digital filtering was performed to avoid phase shift. Eventually, each sEMG signal was min–max normalized within each subject and for each muscle, thus mapping the values in the [0–1] interval. An example of normalized envelopes in a representative stride is reported in Fig. 5. Foot-switch signals were processed for identifying the different gait cycles and phases (stance and swing), according to the approach discussed in [37]. The study [37] describes and validates an algorithm for the segmentation into separate gait cycles of the basographic signal, measured by means of three foot-switches during walking. The algorithm is able to provide also the four main gait sub-phases: heel contact (H), flat foot contact (F), push off or heel off (P), swing (S). Based on this segmentation, the algorithm classifies the different gait cycles characterized by a specific sequence of gait phases (HFPS, PFPS, FPS and so on).

### Data preparation

**Fig. 6:**
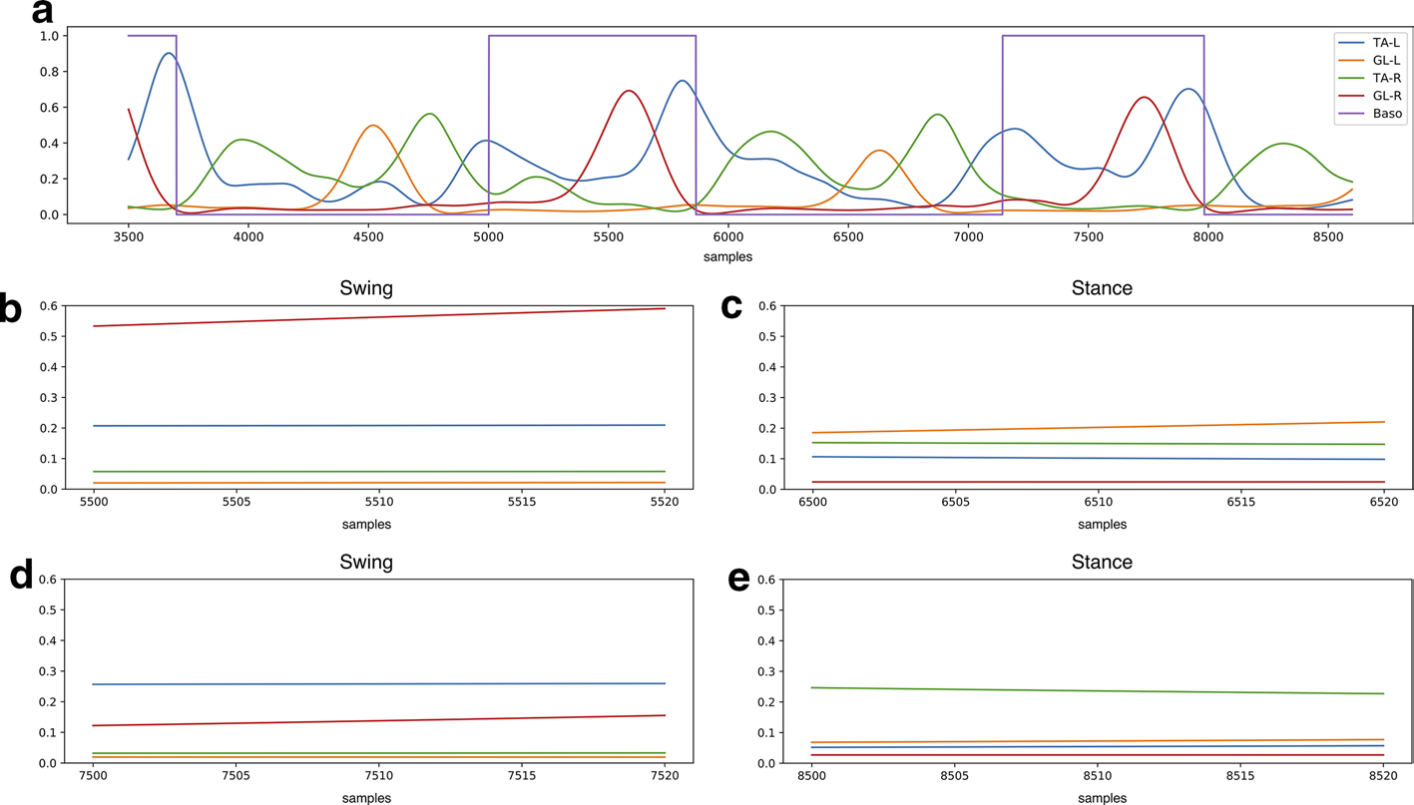
**a** Example of a normalized full-wave rectified and enveloped signal from left (blue line) and right (green line) tibialis anterior and left (orange line) and right (brown line) gastrocnemius lateralis, superimposed to left basographic signal (violet line), in two strides of a representative subject. **b**, **d** Two representative 20-sample windows taken from the swing phases of the signal depicted in **a**. **c**, **e** Two representative 20-sample windows taken from the stance phases of the signal depicted in panel a)

In our experiments, the neural network classifier is fed with the envelope of the EMG signals, thus the classification is based on hidden features automatically learned from the network. As demonstrated in [12], this approach provides better performances than using hand-crafted time-dependent features. For suitably training the classifier, each sEMG signal was split into 20-sample windows (corresponding to 10 ms). Examples of 20-sample windows taken from two consecutive steps of a representative subject are depicted in Fig. 6. A chronological sequence of 200-sample vectors was created, where each vector included the ten synchronized 20-sample windows from the sEMG signals of the ten muscles (five for each leg). In details, the first sample of the first 200-sample vector of the sequence was the first sample of the EMG signal from the muscle 1 (TA, right leg), the second sample of the first 200-sample vector was the first sample of the EMG signal from the muscle 2 (GL, right leg), and so on up to the tenth muscle (RF, left leg). Then, a specific label was assigned to each 200-sample vector as follows: if the value of all the samples of the basographic signal corresponding to the 200-sample vector was 0 (or 1), a global label 0 (or 1) was assigned to the 200-sample vector. 200-sample vectors including swing-to-stance or stance-to-swing transitions were not considered during phase classification, as the goal was to train the network to distinguish between stance and swing phases. On the contrary, all the windows, including those corresponding to a phase transition, were used to predict the timing of gait events (see “Gait-event identification” section).

### Training the classifier

**Fig. 7:**
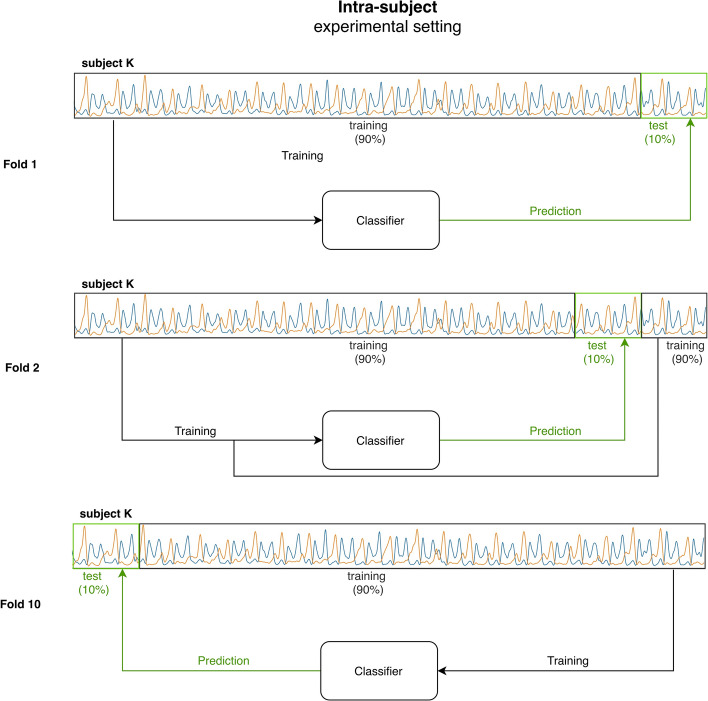
Illustration of the experimental setting used in the intra-subject approach. 90% of the EMG signal of the subject K is used to train the neural network classifier, while the remaining 10% of the trail of the same subject is used for testing phase. For each subject, a 10-fold cross-validation is performed, using, at each fold, a different portion of the subject’s trial for testing. The experiment is performed for all the subjects (*K* = [1, 2,…, N])

**Fig. 8:**
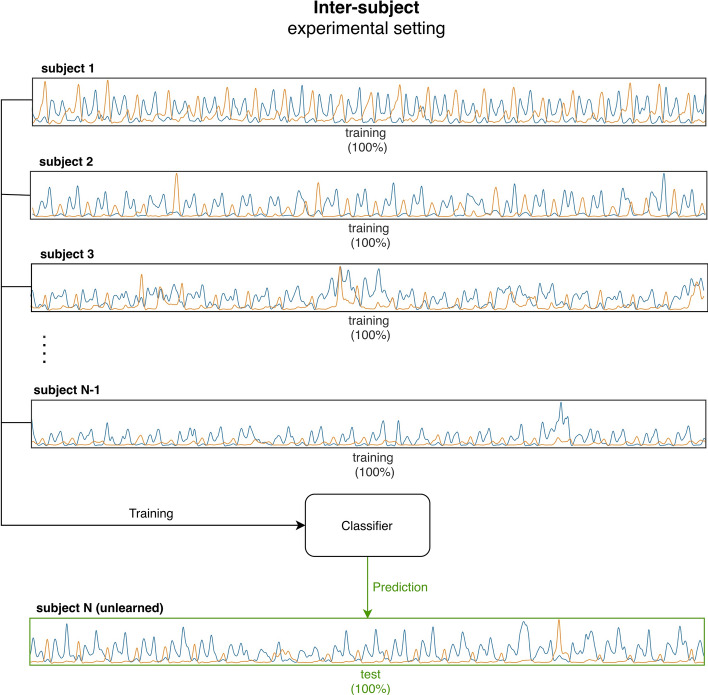
Illustration of the experimental setting used in the inter-subject approach. The EMG signals of N-1 subjects (where N is the total number of subjects) are used to train the neural network classifier. The trained classifier is then used to predict phases from the EMG signal of the remaining (unlearned) subject. The experiment is repeated N times, each time using a different subject for testing

Two different approaches for training the classifier were tested and compared: the intra-subject (Fig. 7) and the inter-subject approach (Fig. 8).

#### Intra-subject approach

The approach is based on the attempt at training the Neural Network classifier by means of sEMG data from a single subject and then classifying gait phases in unseen stride sequences of the same subject. To this aim, the signal composed by the chronological sequence of 200-sample vectors characterizing the walking of a single subject was processed as follows: it was split into 10 equal slots of approximately 60,000 samples long each (Fig. 7). Nine out of ten slots were used to train the classifier. All the 200-sample vectors picked up from the nine training slots were provided to the neural network for the training phase. The 200-sample vectors from the remaining single slot were used for the testing phase, considering the corresponding foot-switch signal as ground truth. The procedure was performed ten times, each time using a different slot as test set (tenfold cross-validation). Classification results in each subject were provided as mean value (± standard deviation, SD) over the tenfold. Population (global) results were provided as mean value (± SD) over the 23 subjects.

#### Inter-subject approach

The approach is based on the attempt at training the Neural Network classifier by means of sEMG data from 22 subjects (out of 23 subjects of the present population) and then classifying gait phases in the remaining unseen subject (leave-one-out cross-validation procedure). To this aim, all the 200-sample vectors were picked up from the signals of the 22 subjects and then provided as input to the neural network for the training phase. The 200-sample vectors from the remaining single subject were used for the testing phase, considering the corresponding foot-switch signal as ground truth. The procedure was performed 23 times, each time using a different subject as test set (23-fold cross-validation). Results in each subject were provided as the classification results in a single fold. Population (global) results were provided as mean value (± SD) over the 23-fold. Further details about this approach can be found in [12].

### The neural network

Multi-layer perceptron (MLP) architecture was implemented in the present study. The model was a neural network with one input layer composed of 200 units (corresponding to the 200 signal values forming each window), three hidden layers composed of 512, 256 and 128 neurons, and a one-dimensional output. The output was fed to a sigmoid function and a 0.5 threshold was used to achieve a binary output: when the output of the sigmoid was > 0.5 the label 1 was assigned, otherwise the label 0 was assigned. Rectified linear units (ReLU) were implemented to provide non-linearity between two consecutive hidden layers. In the experiments, stochastic gradient descent was employed as the optimization algorithm and binary cross-entropy as the loss function. Learning rate was experimentally set to 0.01. Eventually, MLP model was trained adopting an early stop technique: the network was trained for a maximum of 100 epochs, stopping when the accuracy on the validation set (composed of the last 10% of subject strides) did not increase for 10 consecutive epochs. We stored the network weights learned at the epoch providing the best accuracy on the validation set and then used such a trained model to evaluate performances on the test sets.

### Gait-event identification

The foot–floor-contact signal was predicted by chronologically arranging the binary output of MLP network. A vector was provided as output, where sequences of 1 (swing phase) alternate with sequences of 0 (stance phase). Literature reported that stance and swing phase during healthy walking at typical speed last on average 60% and 40% of gait cycle [38]. Starting from this observation, all the transitions in the foot–floor-contact signal lasting less than 175 ms were rejected (≈ 16% of gait cycle). During the post-processing of the predicted basographic signal, 180 spikes (false phases) per subject were removed on average. Average spike duration was 7.8 ± 32.8 samples, corresponding to 3.9 ± 16.4 ms. Then, gait events were identified in the cleaned signal. Swing-to-stance transitions (heel-strike, HS) were assessed as the sample when the sample value switched from 1 to 0. In the same way, stance-to-swing transitions (toe-off, TO) were assessed as the sample when the sample value switched from 0 to 1. Performance of predictions was provided in terms of precision, recall, and F1-score. Precision is defined as

1 $$ {\text{Precision}} = \frac{TP}{TP + FP} $$

where *TP*s are true positives and *FP*s are false positives. Recall is defined as

2 $$ {\text{Recall}} = \frac{TP}{TP + FN} $$

where *FN*s are false negatives. F1-score is defined as

3 $$ F1 - {\text{score}} = 2 \cdot \frac{{{\text{Precision}} \cdot {\text{Recall}}}}{{{\text{Precision}} + {\text{Recall}}}}. $$

A predicted HS or TO at time *t*_*p*_ was acknowledged as true positive (*TP*) if an event of the same type occurs in the ground truth signal at time *t*_*g*_ such that $\left| {t_{g} - t_{p} } \right|$ < T. T is a temporal tolerance, set to 600 ms. Otherwise, the predicted event was acknowledged as a false positive (*FP*). For all the true positives, mean absolute error (*MAE*) was computed as the average time distance between the predicted event and the corresponding one in ground truth signal.

### Statistics

To test the significance of the difference of data distributions (*MAE*, precision, recall, F1-score) between intra-subject and inter-subject approaches, statistical tests were performed. First of all, the normality of each data distribution was evaluated by means of Shapiro–Wilk test. Then, two-tailed, non-paired Student’s *t* test was performed to evaluate the significance of the difference between normally distributed samples. In the same way, Kruskal–Wallis test was performed to evaluate the significance of the difference between non-normally distributed samples. Statistical significance for each test was set at 5%.

## Data Availability

sEMG and basographic data analyzed in the present study are going to be published in a public dataset, and are currently available for research purposes by contacting the authors.

## Abbreviations

EMG: Electromyography
FN: False negative
FP: False positive
GL: Gastrocnemius lateralis
HS: Heel-strike
IMUs: Inertial measurement units
MAE: Mean absolute error
MH: Medial hamstrings
MLP: Multi-layer perceptron
ReLU: Rectified linear units
RF: Rectus femoris
sEMG: Surface electromyography
TA: Tibialis anterior
TO: Toe-off
TP: True positive
VL: Vastus lateralis
